# Williams Syndrome With Rare Ureteric Abnormality

**DOI:** 10.7759/cureus.17210

**Published:** 2021-08-16

**Authors:** Jaffar Khan, Khaleel I Al-obaidy, Rong Fan

**Affiliations:** 1 Pathology and Laboratory Medicine, Indiana University School of Medicine, Indianapolis, USA

**Keywords:** william's syndrome, tortuous ureter, connective tissue, cardiovascular, autopsy

## Abstract

Williams syndrome (WS), also known as Williams-Beuren syndrome, is a rare genetic disorder characterized by infantile hypercalcemia, short stature, a varying degree of mental retardation, elfin-like facial features, and cardiovascular abnormalities, including systemic hypertension, aortic hypoplasia, coarctation of the aorta, and valvular heart disease (aortic and pulmonic stenosis, mitral valve prolapsed or bicuspid aortic valve). It is also characterized by friendly and outgoing personality. The majority of WS cases are sporadic, while few are familial. Both sporadic and familial cases are due to deletion of chromosome 7 (7q11.23). Herein, we present an autopsy case of a 16-day-old male infant born to a 25-year-old mother with a history of William syndrome. Prenatal echocardiogram showed supravalvular aortic stenosis and pulmonary stenosis. The postnatal course was complicated by feeding difficulties and desaturation. Gross autopsy findings included generalized edema, macrocephaly with short neck, and multiple facial anomalies (mandibular hypoplasia, depressed nasal bridge, long philtrum, ear malformation, and wide mouth). The heart was hypertrophied with obstructed ventricles and rudimentary, hypoplastic aortic root. An enlarged, dilated, and tortuous left ureter was a unique finding to this case, in addition to variation in the renal arteries' size and an small bowel outpouching located 33 cm from the ileocecal valve. Cytogenetic analysis revealed deletion of chromosome 7 (7q11.23). In conclusion, majority of WS cases are sporadic, and few are familial and are inherited as autosomal dominant.

## Introduction

Williams syndrome (WS) is characterized by typical elfin likefacies, puffiness around the eyes, short nose and full lips, growth delays, mental retardation, extroverted personality, and congenital heart defects such as supravalvular aortic stenosis [1]. The real frequency of this syndrome is unknown, although it is estimated to be 1 in 10,000-50,000 live births [2]. WS is caused by deletion of genes of chromosome 7q11.23, which codes for elastin. It affects 1/10,000 people. Patients with this disease have increased morbidity and mortality, mostly due to cardiovascular complications [3]. The cardiovascular issues could be due to elastin deficiency and increased cellular proliferation in the vessel wall with the subsequent development of obstructive lesions [4].^_. _^Since WS's first description, medical treatment has been refined for the condition, but mental health treatment has not been fully addressed. Therapeutic interventions for language, cognition, and behavior will have to be designed and tested [5].

This article was previously presented as a meeting abstract at the College of American Pathologists 2020 Annual Meeting (CAP20 Virtual) on September 9, 2020.

## Case presentation

**Figure 1:**
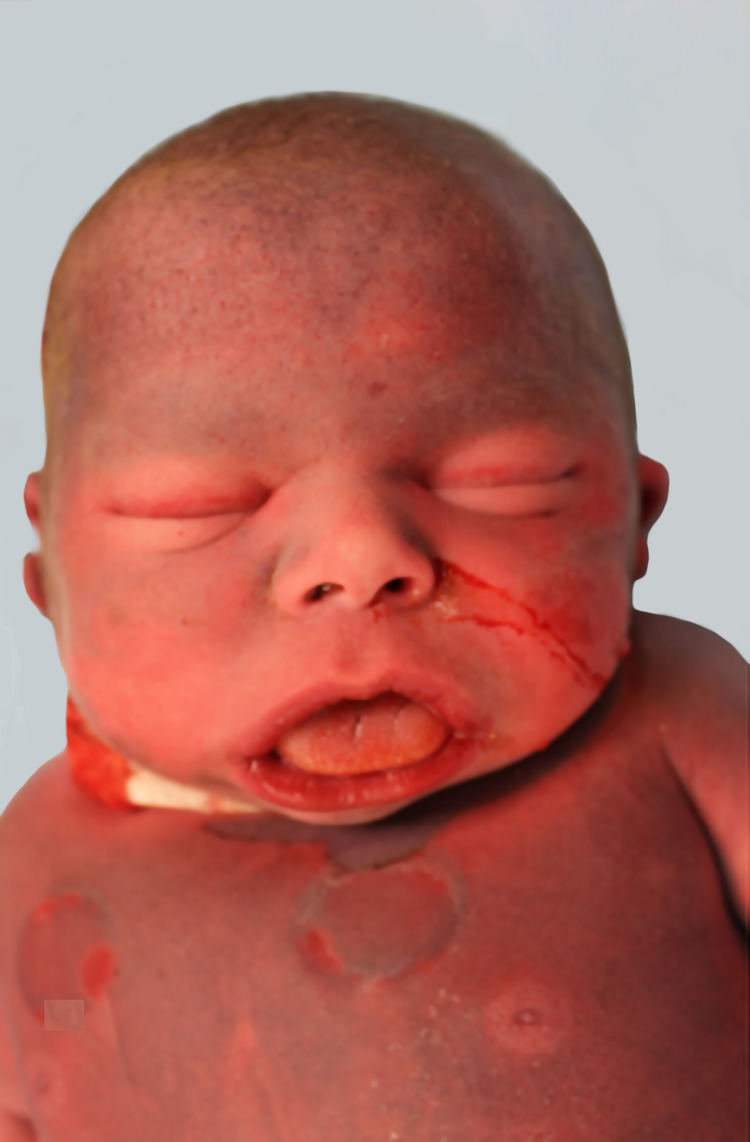
Puffiness around the eyes, short nose

**Figure 2:**
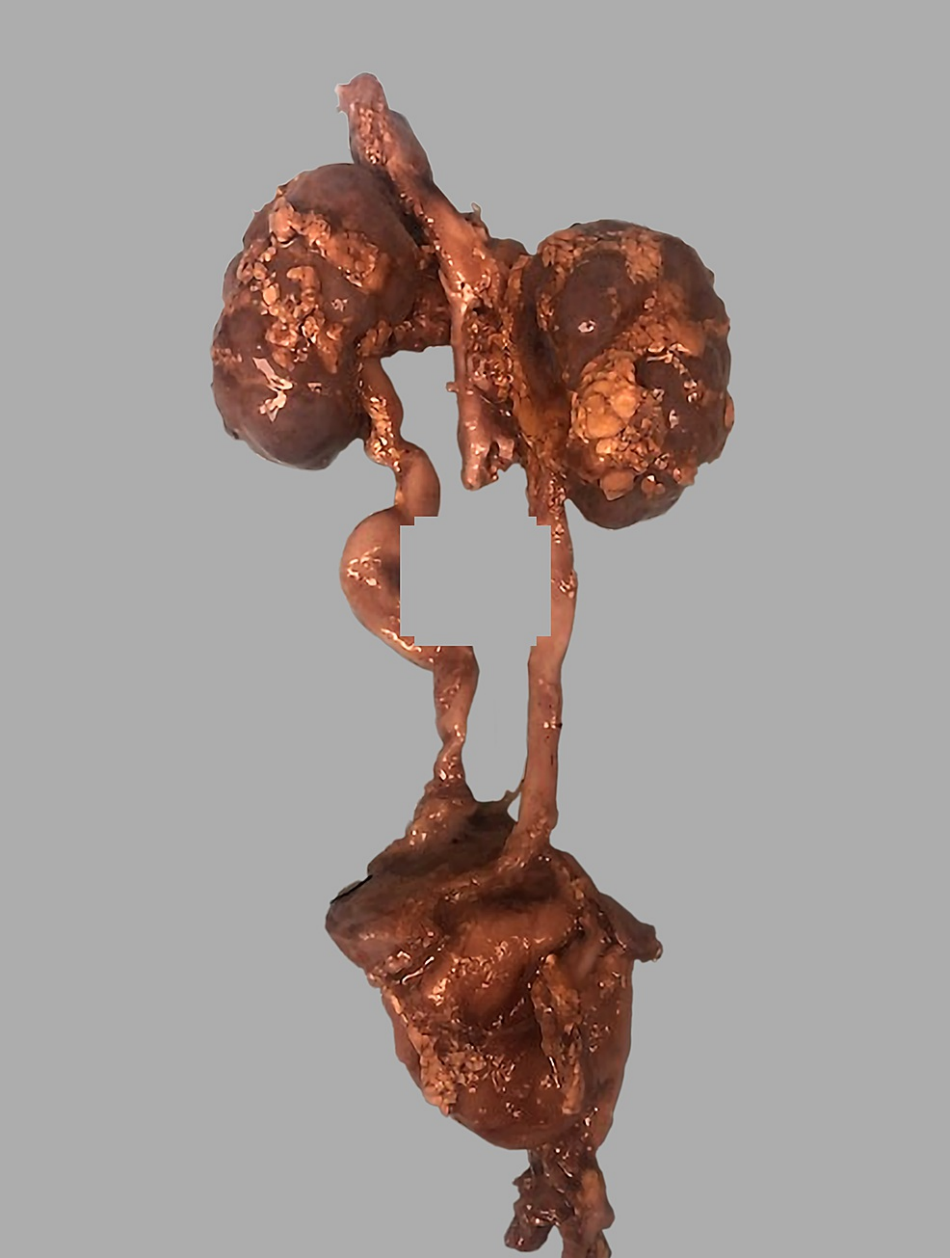
Tortuous ureter

The decedent was a 16-day-old male infant, born at 39 weeks (clinical gestational age determined by second prenatal ultrasound) via cesarean section with no labor complications, to a 25-year-old female. The decedent's mother had history of WS. The mother had appropriate prenatal care prior to delivery and had history of hypertension, asthma, and obesity. Prenatal echocardiogram showed supravalvular aortic stenosis and branch pulmonary stenosis. Apgar score at 1 and 5 minutes was 6 and 8, respectively. The initial saturations were below expected levels and he was placed on continuous positive airway pressure (CPAP). The patient was then transferred to the neonatal intensive care unit. Cytogenetics of the decedent showed deletion of 7q11.23, which is associated with WS. He was weaned off of CPAP but he continued to have feeding difficulties and most of his feeding was through nasogastric tube. On day 14 his heart rate dropped to 50 bpm. He was then intubated and chest compressions were started but unfortunately the patient passed away. Autopsy gross examination revealed significant dysmorphology externally. Dysmorphological evaluation revealed generalized body swelling (anasarca), macrocephaly with shortened neck, hypoplasia of the mandible, depressed nasal bridge, fullness of the midface, long philtrum, wide mouth, puffiness around the eyes and lips, malformed external normally set ears, and shortened and hypertrophied tongue (Figure 1). Along with these features the decedent cytogenetics test showed 7q11.23 gene deletion, which is associated with WS. The heart weighs 34.2 g (expected 20.4 g). The great vessels entered and exit the heart in the usual manner, and a normal innominate vein was identified. The epicardial coronary arteries were normally distributed, and the right and left coronary arteries were poorly visualized. The aortic arch was left-sided with the usual branching pattern. There were two extracorporeal membrane oxygenation lines in the vessels of the neck (carotids). There was narrowing of aorta before the aortic arch. There was hypoplasia of the aortic root, and the aortic valve was very rudimentary, suggesting of supravalvular aortic stenosis. The ductus arteriosus was widely patent as expected. The heart was opened in the direction of blood flow. The cardiac chambers were normally related, there was left ventricular and right ventricular hypertrophy, and the right and left ventricular cavities or chambers were very small due to hypertrophied ventricles. There were no atrial or ventricular septal defects. The foramen ovale was normally formed, and the flap valve of the foramen ovale appears competent. The cardiac valves were unremarkable. The important findings were an enlarged tortuous left ureter (Figure 2).

## Discussion

The main complication in WS is the obstructive cardiac lesions that result in outflow obstruction. These lesions can be repaired with aortic valve replacement and has been successful even in the middle-aged patients with this diagnosis[6].The majority of the patients present with developmental delay and two-thirds of the patients surveyed had cardiac murmur [7]. The condition is associated with a range of linguistic, cognitive, and social issues. The impact of these difficulties becomes evident when this group of patients age, and this increases the risk of social exclusion [8]. Very limited research exists on the educational provision and academic achievements of children with WS, including the non-existing literature on their voices, and doing more research in this area can improve the education and its outcomes [9]. WS is a multisystem disease and cardiac abnormalities are the leading cause of morbidity and mortality in these patients. Cardiovascular abnormalities are the major determining factor in the trajectory of these patients and advances in surgical techniques can improve prognosis [10]. These patients also have tendency to develop hernias, which suggests that they may have some kind of connective tissue disorders [11]. Molecular and genetic studies have indicated that elastin deficiency may be the culprit for cardiovascular and other connective tissue defects in this group of patients [12]. Identifying the genetic alteration pathways and targeting those can change the course of the disease and improve prognosis in these patients [13]. In our case the patient had severe cardiovascular abnormalities. Prenatal echocardiogram showed supravalvular aortic stenosis and pulmonary stenosis. The postnatal course was complicated by feeding difficulties and desaturation. Grossly the heart was hypertrophied with obstructed ventricles and rudimentary, hypoplastic aortic root, indicating the primary cause of mortality in the patient. Gross autopsy findings included generalized edema, macrocephaly with short neck, and multiple facial anomalies including mandiblular hypoplasia, depressed nasal bridge, long philtrum, ear malformation, and wide mouth. An enlarged, dilated, and tortuous left ureter was a unique finding to this case, in addition to variation in the renal arteries' size and a small bowel outpouching located 33 cm from the ileocecal valve.

## Conclusions

In conclusion, a majority of WS cases are sporadic, and few are familial and are inherited as autosomal dominant. Ureteric abnormality seen in this case is extremely rare and it is unclear whether this constitutes a defining feature of this syndrome or not, but worth investigating. Progress in understanding the complex developmental role of the extracellular matrix will likely result in new therapies for the connective tissue abnormalities.
